# Recreational Screen Time Behaviors during the COVID-19 Pandemic in the U.S.: A Mixed-Methods Study among a Diverse Population-Based Sample of Emerging Adults

**DOI:** 10.3390/ijerph18094613

**Published:** 2021-04-27

**Authors:** Brooke E. Wagner, Amanda L. Folk, Samantha L. Hahn, Daheia J. Barr-Anderson, Nicole Larson, Dianne Neumark-Sztainer

**Affiliations:** 1 School of Kinesiology, University of Minnesota, Minneapolis, MN 55455, USA; folk0039@umn.edu (A.L.F.); barra027@umn.edu (D.J.B.-A.); 2 Division of Epidemiology & Community Health, University of Minnesota, Minneapolis, MN 55455, USA; hahn0203@umn.edu (S.L.H.); larsonn@umn.edu (N.L.); neumark@epi.umn.edu (D.N.-S.); 3 Department of Psychiatry & Behavioral Sciences, University of Minnesota Medical School, Minneapolis, MN 55455, USA

**Keywords:** COVID-19, screen time, mental health, qualitative, emerging adults

## Abstract

Understanding how screen time behaviors changed during the COVID-19 pandemic is important to inform the design of health promotion interventions. The purpose of this study was to quantify and describe changes in recreational screen time from 2018 to 2020 among a diverse sample of emerging adults. Participants (n = 716) reported their average weekly recreational screen time in 2018 and again during the pandemic in 2020. Additionally, participants qualitatively reported how events related to COVID-19 had influenced their screen time. Weekly recreational screen time increased from 25.9 ± 11.9 h in 2018 to 28.5 ± 11.6 h during COVID-19 (*p* < 0.001). The form of screen time most commonly reported to increase was TV shows and streaming services (n = 233). Commonly reported reasons for changes in screen time were boredom (n = 112) and a desire to connect with others (n = 52). Some participants reported trying to reduce screen time because of its negative impact on their mental health (n = 32). Findings suggest that screen time and mental health may be intertwined during the pandemic as it may lead to poorer mental health for some, while promoting connectedness for others. Health professionals and public health messaging could promote specific forms for screen time to encourage social connection during the COVID-19 pandemic and beyond.

## 1. Introduction

Sedentary behavior is suggested to have negative impacts on overall health that are independent of physical activity level, including increased risk of all-cause and cardiovascular mortality, type 2 diabetes, and poorer dietary intake [1]. Beyond physical health impacts, greater time spent engaging in sedentary behavior is also associated with poorer mental health outcomes such as anxiety and depression [2]. Unfortunately, the amount of time U.S. adults are engaging in sedentary behavior has increased significantly over time [3]. As a result, the 2018 physical activity guidelines for Americans added an aim to reduce sedentary time by replacing it with more active behaviors [4]. Recently, more attention has been placed on context of sedentary behavior, especially screen time (i.e., mentally active versus passive) and associated health outcomes. There is growing research that supports that passive sedentary behavior and screen use, such as leisure time activities including television (TV) watching, is more harmful to both physical and mental health than mentally active sedentary behavior and screen use such as occupational contexts, reading, or socializing with others [5,6,7,8]. Therefore, both the quantity and type of sedentary time are important in understanding health implications.

Recreational screen time, which often includes watching TV, computer use, and smartphone use, is one of the most common means of engaging in sedentary behavior [9]. There is some evidence to suggest lifestyle behaviors, such as recreational screen time, have been altered as a result of physical distancing measures put in place during the COVID-19 pandemic [10,11,12]. Considering the potential mental and physical health implications of passive sedentary behavior and screen time [5,13,14,15,16], monitoring and understanding trends in screen time during COVID-19 is important to understanding overall health impacts of the pandemic. Emerging adults are of particular interest when assessing screen time since poor health behaviors adopted during these life stages may persist into adulthood and increase the risk of future chronic disease [17]. However, forms of recreational screen time that have changed during the pandemic have not yet been explored in this population. Given that sedentary behaviors, particularly screen time, are behaviors that have shown progressive, secular increases over time [3], it is likely that screen time will remain elevated even after COVID-19 restrictions are lifted. Thus, an improved understanding of ways in which emerging adults are utilizing screens can further inform interventions aimed at minimizing screen time or encouraging more active screen time behaviors during the pandemic and beyond.

Although there is growing evidence that screen time use has increased in the overall population during the pandemic [12], little is known about emerging adult populations or the specific forms of change in screen time (i.e., mode of screen use or content). Therefore, the purpose of this paper is to utilize a mixed-methods approach to explore how events related to COVID-19 have influenced patterns in recreational screen time and reasons for changes in an ethnically/racially and socioeconomically diverse sample of emerging adults.

## 2. Materials and Methods

### 2.1. Study Design and Sample

The C-EAT (COVID-19 Eating and Activity over Time) study invited participants in the EAT 2010–2018 longitudinal cohort to complete an online survey in 2020 during the U.S. outbreak of COVID-19. Participants in EAT 2010–2018 included a population-based sample of young people who attended middle school or high school in Minneapolis-St. Paul, Minnesota in 2009–2010, and were followed over time [18,19,20]. The C-EAT survey was designed to capture changes in eating and activity behaviors and markers of psychosocial well-being during COVID-19 [21,22]. Email, text message, and mailed invitations were sent during the months of April to October 2020 to the 1568 emerging adults who had completed the most recent follow-up survey in 2017–2018. Responses were received from 46% of the sample (n = 720), of which a subsample of 716 had reported on whether or not they believed COVID-19 influenced their recreational screen time and were ultimately included for final screen time behavior analyses. Informed consent was obtained from all participants and protocols were approved by the University of Minnesota’s Institutional Review Board.

Of the full sample (n = 720), 62% were female, 29.6% were White, 23.9% were Asian American, 18.2% were Black or African American, and 16.5% were Hispanic or Latino. The mean age of respondents was 24.7 ± 2.0 years. Approximately one third of the sample (32.7%) were considered of low socioeconomic status based on report of parental educational attainment at baseline (Table 1).

### 2.2. Survey Measures

*Recreational Screen Time* was assessed at EAT 2018 and C-EAT using measures modified from previous surveys, including prior waves of the EAT study [23,24,25]. The validation of similar items has been described elsewhere [26]. Two similar questions were asked at each time point to ascertain weekday and weekend day recreational screen time use, with the C-EAT survey asking participants to think about screen time specifically within the past month during COVID-19: “In the past month, on an average [week or weekend] day, how many hours of recreational screen time (for example, television, computer, social media, video games, smartphone or tablet) did you have a day? Do not include activities you did for work or school.” Response options included: “0 h a day,” “1/2 h a day,” “1 h a day,” “2 h a day,” “3 h a day,” “4 h a day,” and “5+ hours a day.” The response options were coded to match the corresponding hours, with the 5+ category coded as 6 h for analysis. Weekday and weekend responses were combined to determine a weighted weekly average of hourly screen time for each time point. Test-retest reliability was examined at EAT 2018 using data from 112 participants who completed the survey twice over 3 weeks (*r* = 0.76).

*Perceived Influence of COVID-19 on Recreational Screen Time* was assessed as part of the C-EAT survey by asking, “Have recent events related to COVID-19 influenced your media use?” Response options included “No,” “Yes, somewhat,” or “Yes, very much.” If participants responded with either of the yes responses, they were directed to the following open-ended question: “Please comment on how events related to COVID-19 have influenced your media use, including use of social media, watching shows or movies, playing video or computer games. What event(s) related to COVID-19 have been the most important to your media use?”

*Sociodemographic Variables* were self-reported. As part of the baseline survey in 2009–2010, participants identified their ethnicity/race (White, Black or African American, Hispanic or Latino, Asian American, Native Hawaiian or other Pacific Islander, American Indian or Native American, or Other) and responded to questions relating to household socioeconomic status (SES), which was primarily determined by the highest education level of the participant’s parent(s). Additional variables used to determine SES included family eligibility for public assistance, adolescent eligibility for free or reduced-price school lunch, and maternal and paternal employment status [27,28]. Sociodemographic information collected as part of the C-EAT survey included birth year (used to estimate age at time of C-EAT) and gender identity (male, female, or different identity).

### 2.3. Statistical Analysis

*Quantitative.* Descriptive statistics were generated for all sociodemographic variables, as well as measures of the amount of recreational screen time. Paired t-test analyses were utilized to compare recreational screen time in 2018 to screen time in 2020 during the COVID-19 pandemic. A one-way analysis of variance (ANOVA) was utilized to determine differences in change in recreational screen time between demographic groups. All analyses were conducted using SAS 9.4 software (SAS Institute, Cary, NC, USA, 2015).

*Qualitative.* A modified grounded theory approach was used to assign codes and identify themes of the open-ended responses [29]. This method for analyzing qualitative data involves describing, organizing, connecting, corroborating, and representing the information gathered from participants. All comments were initially read by two independent researchers who worked separately to identify preliminary codes to later organize participant responses using a spreadsheet. Next, the two independent researchers compared preliminary codebooks to finalize, then both researchers independently coded each comment; codes were not mutually exclusive, meaning participant responses could indicate more than one code. Discrepancies in the response coding were resolved through consensus. Percentages presented in the qualitative section of the results are based on the sample of participants who responded to the survey question asking if COVID-19 had affected their media use with either “Yes, somewhat” or “Yes, very much,” regardless of whether they expanded further (n = 545).

## 3. Results

### 3.1. Quantitative Changes in Recreational Screen Time

Recreational screen time increased by 2.6 ± 13.5 h per week from 2018 to 2020, going from an average of 25.9 ± 11.9 h per week in 2018 to 28.5 ± 11.6 h per week during the pandemic (*p* < 0.001). Change in screen time was not related to gender identity (*p* = 0.57), ethnicity/race (*p* = 0.29), or socioeconomic status (*p* = 0.14). Over 76% of participants (n = 545) reported that they perceived their recreational screen time had been influenced at least somewhat by the COVID-19 pandemic. Nearly half of the participants (48.6%) reported an increase in average weekly hours, 32% reported a decrease, and 19.4% reported no change in screen time. The magnitude of increase and decrease in change in screen time is presented in Table 1.

### 3.2. Forms of Change in Recreational Screen Time

**Figure 1:**
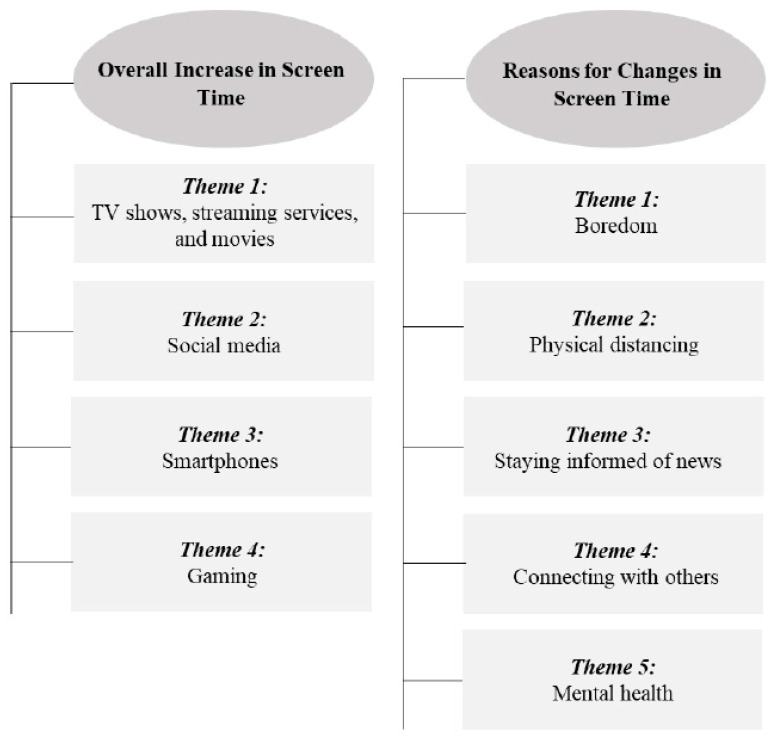
Main themes emerging from qualitative data on change in screen time behaviors.

After analyzing the results with regard to perceived changes in screen time and reason for those changes, four themes emerged related to an increase in screen time, and five themes emerged related to reasons for the changes in screen time (Figure 1).

#### 3.2.1. Overall Increase in Recreational Screen Time

A major theme among comments regarding the perceived influence of events related to COVID-19 on recreational screen time, was an overall increase in use of different forms of screen time. Four themes surfaced related to the forms of screen time that were impacted the most: (1) TV shows, streaming services, and movies, (2) social media, (3) smartphones, and (4) gaming.

**TV shows, streaming services, and movies.** The most common theme was that watching TV shows, streaming services (e.g., Hulu, YouTube, Netflix, etc.), and movies had increased during the pandemic (43% of participants, n = 233).

*“I also am watching TV on streaming services much more than I was in the past. Usually I would watch TV once a week or even less, now I watch TV almost every day.”*

*“I’ve watched almost every show on Netflix. I’ve actually also had to download Hulu and am now looking for another app.”*

**Social media.** Participants (37%; n = 204) also reported an increase in social media use. Participants reported specific types of social media such as Facebook, Twitter, TikTok, and Instagram, as well as an increase in social media in general.

*“Social media became more present and I would often find myself just browsing through social media.”*

*“I’ve been on social media a bit more than I usually would because I have more time to go on it. There hasn’t been too [much] to do to fill my hours after work.”*

**Smartphones.** A specific increase in time spent using smartphone screens was reported by 20% of participants (n = 111), as another common theme related to the influence of COVID-19 on screen time.

*“Because I have been stuck inside more, I have been on my phone almost non-stop since quarantine was started.”*

*“I use my phone about 6 h a day! I normally try to use my phone ~3 h a day. I read the news, check social media, watch videos, and talk to my friends, all more than normal. I’ve also started playing games which I don’t normally do on my phone.”*

**Gaming.** Participants (17%; n = 94) also described an increase in gaming on electronic devices. Multiple platforms for gaming were reported, including phone games, computer games, and video game consoles.

*“Trying to play video games that [can] involve others or play ‘online’ with others was a huge part of my time in April/May when Animal Crossing came out for the Switch.”*

*“Video games [have] been a great stress relief outlet for me. I can play online with my friends and we chat with headsets/microphones. The multiplayer aspect allows me to socialize regularly.”*

#### 3.2.2. Reasons for Changes in Use of Recreational Screen Time

Nearly 55% (n = 299) of participants reported a reason for their change in recreational screen time due to the COVID-19 pandemic. Several different reasons were identified, and five themes emerged: (1) boredom, (2) physical distancing, (3) staying informed of news, (4) connecting with others, and (5) mental health.

**Boredom.** The most common theme that was mentioned as a reason for change in emerging adults’ use of screens was boredom, having free time to fill, or feeling like there was nothing to do. This theme was reported by 21% of participants (n = 112).

*“I find myself bored and looking to waste time on my phone.”*

*“Since there isn’t much to do, I have spent some of my time watching TV [shows] or on social media.”*

**Physical distancing.** The use of screens as a result of the stay-at-home order was mentioned by 18% of participants (n = 99). Participant responses were categorized here if they explicitly mentioned the stay-at-home order, quarantine, shelter in place, the inability to see family and friends due to physical distancing, or the inability to go out as a reason why their use of screens had changed since the start of COVID-19.

*“Being stuck inside the house since March has made me consume a lot more media.”*

*“Since the stay at home order, I have been on my phone and PS4 to pass the time with little else to do to keep entertained.”*

**Staying informed of news.** Wanting to stay informed on the current situation and news stories related to the COVID-19 pandemic was another popular theme reported by 17% of participants (n = 95). This theme also included general news-related media where the type of news was not specified in the answer.

*“I used it to stay up to date on COVID current news like spikes in cases and social activism.”*

*“Using my phone to stay up-to-date on the progression of the pandemic has also increased my media use. I would never actively seek the news, but now I find myself doing that a lot more.”*

**Connecting with others.** Participants (10%; n = 52) discussed using social media and other online platforms as a means to connect with friends and family and stay engaged in human interaction since they did not have the opportunity to see friends and family in person.

*“I have spent much more time on social media to stay connected with friends, family members, and professors.”*

*“We are now relying on technology to stay connected with our families. I video chat with my family/friends a few times a week…”*

**Mental health.** Some participants mentioned they were making an effort to avoid screen use because of mental health concerns (6%; n = 32).

*“…because COVID is so present in social media, I avoid it when I feel very drained or anxious.”*

*“It’s actually decreased my social media use a little bit. I’m finding that all the negative news between COVID and the police brutalities that I’m in a more positive state when I limit my use of social media.”*

## 4. Discussion

The aims of this mixed-methods study were to quantify and describe changes in recreational screen time behavior of emerging adults during the COVID-19 pandemic. On average, there was a significant increase in the hours of recreational screen time reported during the pandemic compared to two years earlier (i.e., the EAT 2018 assessment). In alignment with this change in screen time hours, a large proportion of participants reported the perception that their recreational screen time was impacted by COVID-19 in some way, although there was individual variability in the magnitude and direction of screen time change. Participants described increases in watching TV and streaming services, social media, gaming, and smartphone use. Participants indicated a number of reasons that COVID-19 impacted their screen time, including a desire to stay more connected with family/friends, staying updated on news, physical distancing, and boredom. Some participants also reported a desire to decrease their screen time due to resulting mental health effects.

The increases observed in emerging adults’ screen time in the current study extend the findings of research by Colley et al. (2020) who found that over 50% of adults had increased their screen time on at least two different devices during the pandemic [11]. Further, Meyer et al. (2020) also found that adults over the age of 18 experienced an increase in overall screen time from before to during COVID-19 by as much as 40% [12]. The current study further extends prior findings by showing that the most commonly reported form of recreational screen use increases were for TV shows, streaming services, and movies, followed by social media, smart phones, and gaming. Using a different methodological approach, Colley et al. (2020) also found TV use as well as internet use to have increased among a large percentage of participants during the pandemic.

To expand on the findings from Colley et al. (2020) and Myer et al. (2020), the current study provides a unique opportunity to understand not only ways in which participants have increased their screen time, but also the reasons for those changes by allowing for open-ended responses to survey questions. Although the reasons for change in screen time varied among the current sample, the most commonly reported reason for an increase was the feeling of boredom and seeking to occupy free time. This finding is consistent with literature during pre-pandemic time periods that identify screen time, particularly smart phone use, as a way to reduce boredom [30]. Not only can boredom lead to the use of more technology, but it may also lead to less engagement in hobbies and physical activities [31].

In the same sample used in the current study, 83% of emerging adults perceived their weekly physical activity had been influenced during the pandemic, but nearly 94% experienced a quantitative change in physical activity levels [32]. These data compare with the current screen time behaviors in which less participants perceived their screen time to have been influenced compared with the percentage that changed based on the quantitative data. Additionally, 55.6% of emerging adults decreased their weekly physical activity from 2018 to during the pandemic for various reasons [32]. Parallel to the bi-directional screen time findings, not all participants changed their physical activity levels in the same way. Some participants mentioned barriers to engaging in activity such as the stay-at-home order and the closure of gyms and fitness centers [32]. Additional differences emerged as to how physical activity engagement has changed during the pandemic, similar to the identified differences in changes to screen time. Some changes in activity were in the form of more or less time spent outdoors, being active with others more than before, mental health both positively and negatively impacting physical activity, and some participants reported trying to be more active within their homes and potentially utilizing technology to do so [32]. While nearly 50% of participants in the current sample increased their screen time, and nearly 55% decreased their physical activity, a different sample of adults over the age of 18 revealed that the combination of an increase in physical inactivity and an increase in passive screen use was associated with poor mental health during the pandemic [33]. However, engaging in physical activity can reduce or sometimes even eliminate the negative health effects associated with time spent engaging in sedentary behavior and even passive screen time [7,34], and was also mentioned as a positive benefit to mental health in the current sample [32]. Therefore, our results suggest that interventions aimed at reducing boredom by promoting physical activity, particularly during times of physical distancing, may be needed to not only decrease recreational screen time but also improve physical and mental health outcomes potentially associated with passive screen use.

Additionally, many participants were engaging in screen time to keep up with the news related to COVID-19, revealing that screen use also played an important role in staying informed on important issues during months of rapidly evolving information being reported. The C-EAT study results support a recent review acknowledging the importance of media in the dissemination of information during the pandemic [35]. While staying informed on pertinent information related to the virus and mandates set in place is important for keeping people safe and healthy, a number of participants in the current study reported that general and specifically news-related media had negatively impacted their mental health. Ultimately, these impacts influenced many participants to attempt to reduce their media use and screen time. Since negative consequences for mental health are commonly associated with screen time in general [14,15], and during the pandemic [12,36], future research should examine the use of more mentally active screen time that allows for consumption of necessary media while potentially reducing the associated mental health impact.

Further, the use of screen time and specifically social media may be crucial in favoring social connectedness during times of isolation. In the current sample, staying connected with family, friends, and loved ones was another prevalent reason for changes in screen time. Social connectedness following or during major events or disasters has been used as a crucial coping tool for improving mental health [37], which was specifically true among a group of college students coping with stress during COVID-19 [38]. Because social connection via screen time is considered a type of mentally active screen time, these data suggest that the promotion of screen time use specifically for social connection during isolation may provide a mental health benefit. However, due to the mental health impacts associated with different types of screen time [14,15], more research is warranted to disentangle the potential benefits and harms of screen time and social media use at a time when it is recommended that in-person communication be limited.

Study strengths and limitations should be considered when interpreting the findings. The diversity of the sample is a study strength and allows us to better understand how emerging adults with diverse ethnic/racial identities have modified their recreational screen time during the pandemic. Further, the use of longitudinal data, in addition to the qualitative responses in C-EAT, allow for building understanding of both actual and perceived changes that have occurred in screen time behaviors. To our knowledge, this is the first study to not only quantify changes in screen time using a prospective cohort study, but also qualitatively examine the perceived influence of COVID-19 on screen time. Overall, this information is timely in regard to the response to the pandemic and health promotion efforts during this period when many states across the nation are maintaining limits on social gatherings to reduce the transmission of COVID-19.

Although ample strengths of this study exist, it is not without limitations. First, many participants only addressed the first part of the open-ended question about what changed regarding their media use habits, but did not address the second part of the question which aimed to understand why these changes occurred as a result of the COVID-19 pandemic. Second, the data used for the quantitative comparison of screen time before and during COVID-19 was based on self-report, which may have resulted in a level of participant reporting bias. Additionally, recreational screen time questions only provided response options of 0 h to 5+ h which limit the ability to capture excessive screen time of 6+ h that some participants may have experienced. The measure may also not be sensitive enough to capture smaller changes in screen time. However, the survey questions used were modified from validated measures [23,24,25,26] and were found to have high test-retest reliability. Another limitation is that the C-EAT survey was fielded during spring, summer, and early fall, but screen time during the winter months were not assessed. Therefore, the seasonality of the data collection may not have provided a full picture of screen time during all seasons. Lastly, because participants were originally recruited from the Minneapolis/St. Paul area, it is possible that the results may not be generalizable to all emerging adults across the U.S.

## 5. Conclusions

The impact of the COVID-19 pandemic on screen time may be long lasting and the extended time required for an economic rebound may exacerbate the challenges of reducing excess time spent watching TV, gaming, and using social media. The association between negative physical and mental health and excess screen time are of ongoing public health concern. Therefore, the variation in types and reasons for the increases in screen time (i.e., mentally active or passive) are important for informing future interventions aimed at reducing screen time even after the magnitude of the pandemic and need for restrictions on social gatherings is reduced. Additionally, although screen time generally increased during the COVID-19 pandemic in the current sample, positive implications should also be considered. It is important to note that screen time did promote some mentally active screen behaviors such as social connectedness and the ability to stay engaged and informed on topics related to the rapidly evolving pandemic. Future research should further explore the benefits of screen time and social media use during times of isolation not only during the pandemic, but even after the COVID-19 physical distancing restrictions are lifted, and ways to mitigate negative consequences. Levels of screen time will likely remain high beyond the conclusion of the COVID-19 pandemic and this study provides a start in learning not only how public health professionals may be able promote a reduction in screen time behaviors, but how we can also adapt virtual platforms to better support social connection and mental health in the future.

## Figures and Tables

**Table 1:** Participant demographics and screen time behaviors (n = 720).

Variable	Total
Age, Mean (SD)	24.7 (2.0)
Gender, n (%)	
Male	263 (36.5)
Female	447 (62.1)
Different identity	10 (1.4)
Ethnicity/Race, n (%)	
White	213 (29.6)
Asian American	172 (23.9)
Black or African American	130 (18.2)
Hispanic or Latino	119 (16.6)
Other ^a^	85 (11.8)
Socioeconomic Status, n (%)	
Low	231 (32.7)
Low-middle	146 (20.6)
Middle	119 (17.0)
Upper-Middle	131 (18.5)
High	79 (11.2)
Perceived Influence of COVID-19 on media use, n (%)	
Yes, very much	360 (50.3)
Yes, somewhat	185 (25.8)
No	171 (23.9)
Weekly Recreational Screen Time, Mean hours (SD) ^b^	
EAT 2018	25.9 (11.9)
C-EAT	28.5 (11.6) *
Change in Weekly Screen Time from EAT 2018 to C-EAT, Mean hours (SD) ^b^	
Increase (n = 348)	13.1 (8.3)
Decrease (n = 229)	12.4 (8.6)
No Change (n = 139)	n/a

^a^ ”Other” category was collapsed to include Native Hawaiian or other Pacific Islander, American Indian or Native American, and Other. ^b^ Based on analytic sample of n = 716. * Significant increase in hours from EAT 2018 (*p* < 0.001).

## Data Availability

The data presented in this study are not publicly available but can be provided by senior author Dianne Neumark-Sztainer in response to a reasonable request.
